# Autonomous Motivation Is Not Enough: The Role of Compensatory Health Beliefs for the Readiness to Change Stair and Elevator Use

**DOI:** 10.3390/ijerph111212412

**Published:** 2014-11-28

**Authors:** Theda Radtke, Pamela Rackow

**Affiliations:** Department of Psychology, Applied Social Psychology, University of Zurich, Binzmühlestr.14/Box 14, 8050 Zurich, Switzerland; E-Mail: Pamela.Rackow@uzh.ch

**Keywords:** autonomous motivation, compensatory health beliefs, behavior change, readiness to change behavior, stair use, elevator use, physical activity, workplace

## Abstract

Compensatory health beliefs (CHBs) are beliefs that an unhealthy behavior can be compensated with a healthy behavior. In line with the CHBs model, the aim of this study was twofold. First, the study investigated the relationship between autonomous motivation and CHBs that physical inactivity can be compensated by taking the stairs instead of the elevator. Second, the study focused on the associations between CHBs and readiness to use the stairs more often and stair and elevator use. Thus, a cross-sectional online questionnaire was designed that was filled out by 135 participants. Path analysis showed that individuals with stronger autonomous motivation to use the stairs strongly agreed that sedentary behavior could be compensated by taking the stairs instead of the elevator. Moreover, CHBs were positively related to readiness to change behavior, but not to self-reported stair and elevator use. Even though future research is necessary to replicate these findings, autonomous motivation seems to have a positive impact on CHBs which, in turn, might boost an intended behavior change. Thus, promoting possible compensation of physical inactivity might foster the readiness to change the unhealthy behavior.

## 1. Introduction

Individuals make daily decisions about which behaviors to engage in. For example, individuals must decide whether to eat a delicious cake despite the high fat and sugar intake, which might contradict their aim of eating healthily, or whether to use the stairs instead of the elevator to be physically active. Deciding between these opposite behavioral options can induce cognitive dissonance [1]. The interaction between a desire (to carry out an unhealthy behavior, such as using the elevator) and one’s own health goal can produce a motivational conflict [2]. Individuals strive to achieve an ideal balance between fulfilling their desires and pursuing their goals [3]. This search for an ideal balance between maximal pleasure and minimal disadvantage is also called the hedonic principle [4]. A person could, for example, choose to use the elevator instead of taking the stairs because the elevator is a comfortable method of transportation. At the same time, this person pursues the goal of living a healthy life and is aware that taking the elevator is counterproductive for his or her level of physical activity. In this example, cognitive dissonance arises and the dissolution requires self-regulatory processes [2]. One strategy for reducing cognitive dissonance is activating compensatory health beliefs (CHBs; [2]).

CHBs are beliefs that the negative effects of a volitional unhealthy (but pleasurable) behavior can be compensated by engaging in a healthy behavior [5]. To overcome cognitive dissonance, individuals convince themselves that their unhealthy behavior, like taking the elevator at the workplace, is acceptable because they will exercise in the evening or eat healthily in general. In other words, they believe that engaging in healthy behavior will compensate the negative effects of unhealthy choices, such as being inactive at work. CHBs seem to provide the ideal solution, since individuals can choose an unhealthy behavioral option without feeling guilty about having counteracted their goals. Thus, the compensatory behavior is used as a justification for low resistance to an unhealthy behavior [2,5].

A growing body of evidence has shown that CHBs are negatively associated with health behavior change intentions or readiness to change an unhealthy behavior. Behavioral intentions are defined as the subjective likelihood that an individual will engage in a specific behavior [6]. The strength of intentions is measured with Likert scales and the item stem: “I intend to, e.g., exercise for at least …”. In contrast, readiness to change a certain behavior can be defined as the degree to which an individual is ready to change a behavior. Different stages of change are proposed in the Transtheoretical Model [7]. Thus, readiness to change also captures the fact that individuals might not currently consider a behavioral change, because they might not even see the behavior as being problematic. Several methods, including stage categorization and continuous measurements, assess readiness to change. Continuous measures assess attitudes toward changing behavior, in contrast to stage algorithms, which measure plans to change behavior [8]. Researchers have shown that individuals with high CHBs have a lower readiness as well as lower intentions to change an unhealthy behavior such as smoking. Studies have shown that CHBs are negatively associated with readiness to quit smoking [9,10], the intention to be physically active [11], and diet adherence [12].

In addition to the relationship of CHBs with intention and readiness to change, researchers have also shown that CHBs have a negative impact on behavior. For example, CHBs are positively related to poor diabetes self-management and caloric intake [13,14]. Although these findings shed light on CHBs and the association with behavior, little is known about the consequences of CHBs for physical activity. Furthermore, to the best of our knowledge, only one study has investigated the motivational determinants of CHBs [12]. De Ridder [15] emphasized further research on CHBs that consider motivation as an important aspect for individuals who negotiate their personal health goals while facing the tempting, but unhealthy, reality. Thus, in the following, the association of motivation, behavior change, and CHBs is discussed.

### 1.1. Associations between Motivation, Behavior Change, and CHBs

#### 1.1.1. Motivation and Behavior Change

In Self-determination Theory (SDT), Deci and Ryan [16] presume that the motivation for certain behaviors varies along a motivational continuum ranging from intrinsic to extrinsic. Intrinsic motivation indicates a self-determined behavior, meaning that an individual is driven by his or her own interests and experiences enjoyment during the activity. Extrinsic motivation is subdivided into three motivational styles: identified, introjected, and external regulation. Intrinsic and identified regulation can be combined to form autonomous motivation. Extrinsic and introjected regulation can be combined to form controlled motivation [17,18]. Autonomous motivation as a prototype of self-determined behavior is based on the satisfaction experienced for the behavior itself. In comparison, controlled motivation is characterized by satisfaction based on reinforcement. Individuals with autonomous motivation for certain behaviors freely engage in the behavior due to interest, personal convictions, or enjoyment. Controlled motivated individuals, in contrast, act because of feelings of guilt or anxiety, external rewards, or pressure. According to DeCharms [19], autonomous motivation is related to an internal locus of causality when regulating and initiating behavior, whereas controlled motivated individuals perceive the locus of causality as external. As research has shown, being autonomously motivated and having an internal locus of causality are positive predictors of maintained behavior change (for a meta-analysis: [20,21]). In the case of physical activity, individuals with stronger autonomous motivation have stronger intentions to change behavior. In addition, such individuals are more likely to achieve their goals than individuals with controlled motivation [22,23]. Furthermore, prior research in the exercise domain has provided evidence of an association between self-determined motivation and physical exercise (e.g., [24,25]).

#### 1.1.2. Motivation and CHBs

Rabiau *et al.* [2] explained the determinants of activating CHBs and how compensatory intentions are implemented in behavior. In the current study, the focus is CHBs and their determinants. Therefore, only the determinants of CHBs, not the implementation of CHBs in behavior, will be described (for details on implementation, see [2]).

As explained above, CHBs are activated by a motivational conflict between a desire and health goals, which may result in cognitive dissonance. In addition to self-efficacy [2], a major component of this motivational conflict is the extent to which individuals engage in self-set goals out of self-determined motivation (*i.e.*, an individual is motivated to engage in a certain behavior due to strong interest, pleasure, or personal convictions = autonomous motivation) or out of non-self-determined motivation (*i.e.*, an individual is motivated to engage in a certain behavior due to external rewards, pressure, or guilt = controlled motivation). The CHB model suggests [2] that autonomous motivation should decrease the activation of CHBs. The reason is that autonomously motivated individuals should resist temptations that interfere with long-term goals much better than controlled motivated individuals. Autonomous motivation is related to greater behavioral persistence and higher goal attainment. Thus, it is assumed that autonomous motivated individuals will act more consistently with their health aims. In addition, no cognitive dissonance arises that would activate CHBs as a dissonance-reducing strategy. Thus, activation of CHBs is required less for autonomous motivated individuals compared to controlled motivated individuals [2].

A study by Miquelon and colleagues [12] on the motivational determinants of CHBs provided the first evidence that stronger autonomous motivation weakens the activation of CHBs, which, in turn, positively predicted a health behavior change (*i.e.*, weight-loss success). In line with other research [26], stronger autonomous motivation is likely associated with greater resistance against temptations and with higher goal commitment, which reduces the activation of CHBs. In contrast, individuals with low autonomous motivation reported stronger CHBs and less success in weight loss in the study conducted by Miquelon *et al.* [12]. According to these authors, the weak goal attainment was explained by a low capacity to resist temptations that interfere with self-set goals. This in turn activates CHBs to justify the temporary lack of non-adherence to an individual’s goal.

However, little is known about the motivational determinants of CHBs. In addition, research is missing about the consequences of CHBs for physical (in)activity or, more specifically, for the behavior taking the stairs (being physically active) or taking the elevator (being physically inactive).

### 1.2. Physical (In)activity

Taking the stairs instead of the elevator or escalator is one option for increasing physical activity and improving energy imbalance due to sedentary behavior [27]. Boreham, Wallace, and Nevill [28] reported that regular stair use improves cardiovascular outcomes, which was supported by Benn, McCartney, and McKelvie’s [29] findings that stair climbing reaches peak circulatory demands similar to walking uphill or walking on a flat surface and intermittently lifting heavy weights. Therefore, a preference for stair use over elevator/escalator use can improve an individual’s energy balance and result in long-term health benefits, such as weight control [30]. This is important because physical inactivity levels are increasing in many countries. For instance, nearly 60% of the adult Swiss population is insufficiently active or completely inactive, which is comparable to findings in most Western industrialized countries. Physical inactivity is the fourth leading risk factor for global mortality and causes diseases such as breast or colon cancer, type 2 diabetes, and ischemic heart disease [31,32,33].

Several studies have shown that increased stair use is associated with environmental or personal characteristics such as having high accessibility to stairs, visiting a low floor level, having low body mass index (BMI), being male, or not carrying heavy loads [30,34]. In contrast, only a few studies have investigated psychological variables to predict stair use. Results showed that the formation of implementation intentions to use the stairs and the belief that stair use has positive effects on an individual’s weight had an additional effect in explaining stair use [35]. However, more research is needed to examine stair and elevator use with psychological variables. Only by focusing on psychological variables is the development of successful interventions that target increased stair use possible.

### 1.3. Aims of the Study

The purpose of this study was to investigate the association of motivational determinants with CHBs for physical (in)activity. In particular, the aim was to investigate the impact of autonomous/controlled motivation on CHBs. The relationship of CHBs with readiness to change the behavior and self-reported stair and elevator use was examined. These assumptions are based on Miquelon and colleagues’ results [12]. Because the CHB items were formulated so that taking the stairs/being physically active was compensatory behavior to justify physical inactivity, a positive relationship between autonomous motivation to use stairs and CHBs was hypothesized. In contrast, a negative relationship between CHBs and high autonomous motivation to use the elevator was assumed. Additionally, it was proposed that CHBs are positively related to readiness to use the stairs more often. This hypothesis contradicts other research on CHBs (e.g., [9]). However, the CHBs in the present study comprised the investigated behavior (self-reported stair use) as healthy compensatory behavior and not as an unhealthy behavior choice, which was examined in previous research on CHBs. Thus, a negative association between self-reported elevator use and a positive association between self-reported stair use and readiness to use the stairs more often was hypothesized. In sum, individuals with higher levels of autonomous motivation to engage in stair use were suggested to have stronger CHBs and thus, higher readiness to use the stairs more often and higher levels of self-reported stair use.

## 2. Method

### 2.1. Procedure and Participants

The participants were recruited from the employee and student populations of the University of Zurich, a business school, and four companies (a pharmaceutical company, a health insurance company, and two technology companies). These organizations were located in the same office building to ensure that all participants had the same behavioral options between stair and elevator use. In addition, in this office building the stairs and the elevator are located next to each other. Thus, the behavioral decision is independent in terms of the distance from the entrance to the stairs/elevator and the visibility of the elevator/the stairs.

The cross-sectional study was conducted in March and April 2011. Recruitment took place via e-mail lists that advertised the online questionnaire on physical activity habits. On average, 25 min were needed to fill out the online questionnaire. As compensation, participants were eligible to take part in a lottery. The lottery prizes were 12 vouchers for a supermarket worth 60 CHF (US$63) each. The postal addresses of the 12 winners to send out the vouchers were requested in an e-mail sent after the survey was completed.

Overall, *N* = 209 people clicked on the link to the online questionnaire. Of those, nearly one third (*n* = 65) did not answer a question and thus dropped out at the beginning. Nine participants had to be excluded because they stated they had never been in the office building. In sum, the sample for the present study consisted of 135 participants (18.5% were male, 51.1% were female; 30.4% did not indicate gender) with a mean age of 32.23 years (*SD* = 10.19). Twenty-four percent of the sample were university employees, 3.7% were the company employees, 3.4% were PhD students, 29.6% were students, 1.5% indicated another employment status, and 37.8% did not indicate their status.

This study followed the ethical standards of the Declaration of Helsinki [36] and was approved by the checklist of the ethics committee of the Department of Psychology of the University of Zurich in 2011. The companies, the university’s Legal Service, and the business school gave final approval for recruiting participants via the mailing lists. However, participation in the study was voluntary and involved providing confidential responses to the questionnaire. In addition, all individuals were informed by written consent about the study and treated in accordance with the ethical guidelines of the Declaration of Helsinki [36].

### 2.2. Measures

#### 2.2.1. CHBs Scale

Four items (α = 0.84) were adjusted from the original CHBs scale [5] to measure compensatory health beliefs according to sedentary behavior. Items were introduced with a discussion of the benefits and disadvantages of stair and elevator use. Participants rated the extent to which they believed the items on a 6-point Likert-scale ranging from 1 (*strongly disagree*) to 6 (*strongly agree*). The adjustment was based on a German smoking-specific CHBs scale [10], since the study was conducted in German [37]. Items are “If I am less active during the day, I can compensate by taking the stairs,” “If I take the stairs, I can compensate for the negative health effects of prolonged sitting (e.g., in front of the computer),” “I can compensate for too little exercise by taking the stairs every day,” or “I can use the elevator when I move around the whole day.” The last item was recoded before the mean score of the CHBs scale was calculated.

#### 2.2.2. Autonomous and Controlled Motivation

*Autonomous motivation* (according to [38]) was measured separately for using the stairs (seven items) and the elevator (seven items) on a 6-point Likert scale ranging from 1 (*strongly disagree*) to 6 (*strongly agree*). The item stem “If I take the stairs (or the elevator), I take this opportunity because...” was followed by the items like “…I enjoy it” (intrinsic motivation; three items; stair use: *α* = 0.77/elevator use: *α* = 0.60), and “…it is important to me personally” (identified regulation; four items; *α* = 0.89/0.64). *Controlled motivation* was assessed with seven items, separately for stair and elevator use. The item stem “If I take the stairs (or the elevator) was followed by items like “…it feels like a failure if I don’t” (introjected regulation; three items; *α* = 0.74/0.42) and “…others want me to behave like this” (extrinsic regulation; four items; *α* = 0.84/0.84). Following Williams *et al.* [18], the relative autonomy index (RAI) was used to assess the degree of perceived relative autonomy for stair and elevator use. Two indices, one for taking the stairs and one for using the elevator, were calculated with the following formula [17]: (2 × intrinsic subscale + 1 × identified subscale) − (2 × extrinsic subscale + 1 × introjected subscale). The higher the RAI scores, the higher the autonomous motivation reported by the participants.

#### 2.2.3. Readiness to Change Behavior

Readiness to use the stairs more often (according to [39]) was measured with six items (*α* = 0.63), which are closely related to the stages of change within the transtheoretical model [7,8]. In line with continuous measures that correspond to the stages of change (e.g., the University of Rhodes Island Change Assessment (URICA) measure [8]), each item was rated on a 6-point Likert scale ranging from 1 (*strongly disagree*) to 6 (*strongly agree*). Items were “Thinking about using the stairs is a waste of time”, “I believe that I use the stairs to rarely”, “At times I believe that I should use the stairs more often”, “At times it is problematic that I rarely use the stairs”, “I try to use the stairs more often than before”, and “Anyone can think about using the stairs, but I’m really working on it”. Item 1 was reverse-coded for the analyses. Moreover, items were averaged to form a mean value, which means that a higher score on this scale represented a higher level of readiness to change the behavior. Because we assumed that most individuals had never thought intending to take the stairs more often, we chose readiness to change behavior as the measure. Compared to the intention measure, readiness to change behavior measure includes the aspect that an individual had not considered a specific behavior as problematic until now (see Introduction).

#### 2.2.4. Self-Reported Stair and Elevator Use

All participants had to specify how often they took the stairs and the elevator within the last seven days within this specific office building: “How often have you taken the elevator/the stairs in the last seven days?”. Which floor the participants currently spent most of their time was measured to control for effects of the floor level.

### 2.3. Data Analysis

To test the hypothesized model, a manifest path analysis was analyzed with Mplus 7.0 [40]. Due to missing data (28.24%), the full information maximum likelihood (FIML) technique was applied. The FIML estimates model parameters based on all available information for all observed cases. To evaluate the model fit, the *χ*^2^ test was used. According to Bollen and Long [41], the *χ*^2^ should not be larger than two to five times the degrees of freedom. Bootstrapping was used to test the strength and significance of the indirect effects [42].

## 3. Results

### 3.1. Descriptives

In Table 1, the means and standard deviations of all measures are shown. The inter-correlations of the variables are presented in Table 2. CHBs were significantly correlated with autonomous motivation to use the stairs but not with autonomous motivation to use the elevator. The stronger the autonomous motivation to use the stairs, the stronger the CHBs and *vice versa*. As shown in Table 2, individuals who held the compensatory health belief that their sedentary behavior could be compensated through stair use reported having higher readiness to use the stairs more frequently and *vice versa*. However, no correlation between CHBs and self-reported stair and elevator use was found. In addition, women reported having stronger CHBs (women: *M* = 4.16, *SD* = 0.90; men: *M* = 3.42, *SD* = 1.04), *t*(81) = 3.10, *p* < 0.01, *d* = 0.80), as well as a higher readiness to use the stairs more often (women: *M* = 3.50, *SD* = 0.90; men: *M* = 2.97, *SD* = 1.19), *t*(80) = 2.11, *p* = 0.04, *d* = 0.55), compared to men. Age was not correlated with any of the variables and therefore not included in the path model.

**Table 1:** Summary of scale characteristics (mean (*M*), standard deviations (*SD*), and range).

Scale	*M*	*SD*	Range
*CHBs*	4.03	0.99	1.00 to 6.00
*Relative Autonomy Index.(RAI)*			
Stair use	4.37	3.43	–6.00 to 11.67
Elevator use	2.32	3.21	–4.67 to 10.83
*Readiness to change behavior*	3.38	0.99	1.00 to 6.00
*Self-reported behavior (last 7 days)*			
Stair use	3.08	4.55	0.00 to 20.00
Elevator use	5.73	6.34	0.00 to 40.00

Note: CHBs = Compensatory health beliefs.

**Table 2:** Inter-correlations between manifest variables used in the path model.

Scale	1	2	3	4	5	6	7	8	9
1. CHBs	1.00								
2. RAI stair use	0.35 **	1.00							
3. RAI elevator use	–0.13	–0.13	1.00						
4. RCB	0.52 **	0.11	0.08	1.00					
5. SR stair use	0.05	–0.12	0.14	–0.23	1.00				
6. SR elevator use	–0.08	–0.07	–0.19	–0.22	0.29 **	1.00			
7. Floor level	–0.15	–0.14	0.08	0.02	–0.13	0.29 *	1.00		
8. Gender	–0.33 *	–0.12	–0.11	–0.23 *	0.04	0.07	0.12	1.00	
9. Age	0.05	0.19	0.14	0.06	0.09	0.16	0.15	0.39 **	1.00

Notes: CHBs = Compensatory health beliefs; RAI = relative autonomy index; RCB = Readiness to change behavior; SR = Self-reported; Women = 0; Men = 1; * *p* < 0.05; ** *p* < 0.01.

### 3.2. Hypothesized Model

The hypothesized path model specified the associations between autonomous motivation for stair and elevator use separately with CHBs. Furthermore, the indirect association of autonomous motivation with readiness to use the stairs more frequently was specified. In addition, CHBs were hypothesized to be directly associated with readiness to use the stairs more frequently. Last, autonomous motivation (indirect path), CHBs (direct and indirect path), and readiness to use the stairs more frequently (direct path) were defined in relation to self-reported stair and elevator use. All paths were controlled for gender and floor level by including the variables in the regression for CHBs, readiness to use the stairs, and self-reported stair and elevator use.

**Figure 1:**
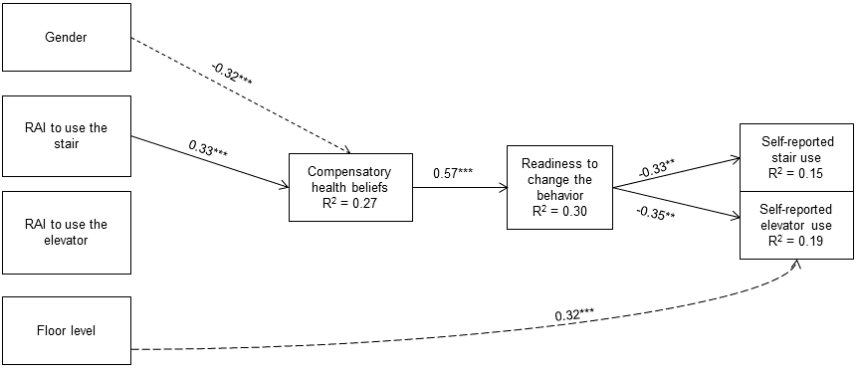
Hypothesized path model.

Notes: Standardized solution. Only significant paths are displayed. However, the RAI to use the elevator was included in Figure 1 to indicate that this measure was part of the model. Dashed lines display control variables; *N* = 5000 bootstrapping resamples; RAI = relative autonomy index; ** *p* < 0.01; *** *p* < 0.001.

The model indicated acceptable model fit (*χ*^2^(22, *N* = 135) = 93.05; *p* < 0.01; CFI = 1.00, TLI = 1.00, RMSEA < 0.01, SRMR < 0.01) because the *χ*^2^ value was not larger than five times the degrees of freedom [41]. In Figure 1, the parameter estimates (standardized solution) are presented. 

CHBs were associated with gender (β = –0.32, *p* = 0.001) and autonomous motivation to use the stairs (β = 0.33, *p* = 0.001) but not with autonomous motivation to use the elevator (β = –0.15, *p* = 0.10). Thus, women had higher CHB values compared to men. Moreover, individuals with stronger autonomous motivation to use the stairs agreed more strongly that sedentary behavior could be compensated by taking the stairs compared to individuals with lower autonomous motivation.

As expected, readiness to change stair use was significantly associated with CHBs (β = 0.57, *p* < 0.001); however, none of the other variables were significantly related to readiness to change stair use. Regarding self-reported stair and elevator use, readiness to change behavior was significantly related to stair (β = −0.33, *p* = 0.01) and elevator (β = −0.35, *p* = 0.01) use. Individuals with higher readiness to use the stairs more often reported that they took the stairs less often. These individuals also reported using the elevator less often.

#### Hypothesized Mediation Analysis

In addition to direct effects, indirect effects were included in the model (the paths are not shown in Figure 1 for clear presentation). First, the mediation of autonomous motivation via CHBs on readiness to change behavior was examined. Results indicated that CHBs mediated the path from autonomous motivation to use the stairs on readiness to change behavior (β = 0.19, *p* = 0.02; 95% CI [0.03; 0.34]). This indirect effect was not demonstrated for autonomous motivation to use the elevator (β = −0.08, *p* = 0.15; 95% CI [−0.20, 0.03]). Second, the indirect effect of autonomous motivation on self-reported stair and elevator use via CHBs was specified. However, the path from autonomous motivation (separately for stair and elevator use) on self-reported stair and elevator use was mediated by neither CHBs nor readiness to change the behavior. Third, whether CHBs were associated with self-reported stair and elevator use via readiness to change behavior was examined. Nevertheless, readiness to change behavior did not mediate the associations between CHBs and self-reported stair or elevator use.

## 4. Discussion

To our knowledge, this study is the first to investigate the relationship between motivational determinants of CHBs and CHBs for a specific physical activity. Moreover, until now no study has examined the association of CHBs with readiness to engage in a compensatory behavior instead of refraining from an unhealthy behavior. Thus, the results make an important contribution to this new area of research. However, there are some methodological limitations, which will be discussed.

### 4.1. Discussion of Results Concerning CHBs

As expected, autonomous motivation to use the stairs was positively associated with CHBs. However, autonomous motivation to use the elevator was not related to the belief that physical inactivity can be compensated by taking the stairs. Even though previous research (e.g., [9,10,11,13,14]) on CHBs found a negative relationship between CHBs and intention to change a behavior, this study yielded the first indication that CHBs are positively related to readiness to use the stairs more regularly. This reverse finding can be explained by the fact that the CHBs in our study contained the intended behavior as the compensatory behavior. Thus, the positive association is reasonable. Next to stair use, the non-significant relationship between autonomous motivation to use the elevator and CHBs is also plausible because the CHB items mainly capture stair use as compensatory behavior instead of elevator use.

In line with previous research [9,11], no relationship was found between CHBs and self-reported stair and elevator use. This result strengthen the assumption that CHBs are rather a motivational construct instead of a volitional construct [43]. The non-significant influence of CHBs on elevator use is also reasonable because the CHBs contained compensatory beliefs mainly regarding stair use. The missing mediation of CHBs via readiness to change the behavior on both behavioral measures confirms Radtke *et al.*’s research [9]. The authors have also shown that smoking-specific CHBs were not mediated via intention on behavior even though Miquelon *et al.* [12] found a significant mediation effect for diet-specific CHBs. Due to the lack of evidence regarding CHBs and different health behaviors, further research is necessary to draw a clearer picture of the relationship between CHBs and readiness/intention to change behavior and the behavior itself.

### 4.2. Discussion of Results for Motivation

Our results showed that autonomous motivation to use the stairs was mediated via CHBs about readiness to engage in stair use. This is in line with Miquelon *et al.*’s findings [12]. For autonomous motivation to use the elevator, neither a direct effect on readiness to engage in stair use nor an indirect effect via CHBs was observed. Thus, motivation for contradictory elevator use seems to have no undermining effect on intended behavior change of stair use. Thus, an intervention that focusses on whether promoting healthy behavior (e.g., stair use) instead of decreasing behavior (e.g., elevator use) is more effective for changing behavior should be investigated (*cf*. [44,45,46,47]). The present study also did not show any indication that autonomous motivation is directly related to behavior. This finding is in line with Miquelon *et al.*’s results [12]. In contrast, readiness to use the stairs more regularly was associated with less stair use. Since the study had a cross-sectional design, individuals with a high readiness to use the stairs reported a low frequency of using the stairs more often and vice versa. However, a significant negative association between readiness to use the stairs more often and elevator use was also found. Thus, it seems that individuals who take the elevator less often compared to those who take it more often tend to be more content to engage in stair use.

### 4.3. Discussion of Results for the Control Variables

Results showed that elevator use was also associated with the floor level the individuals had to visit, which is reasonable. However, the floor level was not the only variable associated with self-reported behavior. Thus, psychological variables such as readiness to change behavior should be investigated. In line with other research, age was not associated with CHBs as well as readiness to change behavior [10]. Nevertheless, gender was associated with CHBs and readiness to change behavior. As expected, men had lower CHBs as well as a lower readiness to change behavior, which is in line with the finding that men are less likely to engage in healthy behaviors (e.g., [48]).

### 4.4. Limitations

Limitations of the study should be considered. The first limitation is the cross-sectional design, which weakens the interpretation of the results in terms of the cause-and-effect relations as well as the mediation analyses. However, Miquelon *et al.* [12] provided evidence for the sequence proposed in this model. Nevertheless, future studies should test the relationship in longitudinal and experimental designs to clarify causal directions. In addition to motivational correlates of CHBs, future studies should examine volitional correlates [9] to get more insight into these beliefs.

Another limitation concerns the self-report of the target behavior. Objective measures of the target behavior are preferred but difficult to realize. Another important point is that the behavior investigated was stair and elevator use in one office building mainly used by a university and four small companies. Thus, the generalization of these results to the general population and other environmental situations is limited. However, the results could be applied to university and academic contexts. The specific inquiry reduced bias in the estimated stair and elevator use because it makes it easier to remember use within a specific building the participants regularly visit or occupy.

Another aspect that requires more investigation is the low internal consistency of several measures. First, the scale introjected regulation for elevator use had a low Cronbach alpha value, which is unacceptable (α < 0.50; (*cf*. [49]). However, this scale was not used as a single measure. Instead, the introjected regulation was one sub-facet of the controlled motivation for elevator use because the RAI Index was used in the following analyses. Using the RAI Index is common practice in SDT research (e.g., [17]. As a consequence of calculating the RAI Index, the low internal consistency of the subscale introjected regulation for elevator use was outbalanced. In order to also discuss possible reasons for a low Cronbach alpha value, it is essential to take the primary purpose of the SDT into account, which is basically providing a framework to study humans’ motivation towards a certain goal [16,20]. When applying this assumption to the present study, “using the elevator” is perhaps not a targeted behavior in nature. This might be one reason that the subscale introjected regulation did not cover all relevant aspects of this particular mode of behavior. Future research is needed to improve and reformulate these items. However, our study is the first step in the right direction, especially since SDT measures should be adapted to the context and area of application in which they are administered (e.g., [16]). This correspondence between item content and target behavior is the precondition for linking the item content to the self-reported target behavior.

Second, the measure readiness to change behavior had a Cronbach alpha value >0.60 and was used as a single measure in the analyses. However, this is in line with other research that reported Cronbach alpha values for process of change measures ranging between 0.62 and 0.80 (e.g., [50]). Nevertheless, the study’s results should be interpreted with caution until the results are replicated.

Additional limitations were the high dropout rate at the beginning of the questionnaire and the high number of missing values. Because the participants were recruited via business e-mail addresses instead of private e-mail addresses, the participants were interrupted while taking part in the study due to work. Furthermore, the high number of missing values especially within the demographic items might be explained by the participants’ requests to be as anonymous as possible. Based on this limitation, another restriction for analyzing data resulted. Due to the small sample size, a path model with manifest mean values instead of latent variables was defined. In contrast to structural equation modeling with latent variables, path analyses assume that all constructs are measured without error. Thus, structural equation modeling with latent variables in larger sample sizes is recommended because it takes measurement errors into account [51].

As the last limitation, the implementation of CHBs in behavior was omitted, although this is part of the CHBs model [2]. However, this study aimed at investigating the relationship of CHBs to a health behavior. Thus, implementing CHBs into action was not of interest but would be a promising approach for future studies.

Taking all limitations together, future research is needed to replicate the findings of this study by taking methodological limitations into account. Otherwise, caution is advised when interpreting the results because this is the first study in this area of research.

## 5. Conclusions

The limitations notwithstanding, the current study provides further insight into the relationship of motivational determinants and CHBs as well as the association of CHBs with readiness to change behavior and the behavior itself. To our knowledge, this is the first study to investigate CHBs for stair and elevator use as a specific facet of physical activity behavior. In addition, this study has provided indications that CHBs might also have a positive impact on readiness to change behavior when CHBs are formulated so that the intended behavior is the compensatory behavior for an unhealthy behavior. Therefore, future research should investigate different kinds of CHBs and their impact on different behaviors since the association between the compensatory behavior and the unhealthy behavior might differ (*cf*. [43]).

The findings might also be relevant for intervention programs. Fostering autonomous motivation for behavior change is advantageous for a positive impact on CHBs as well as on readiness to change behavior (mediated via CHBs). Intervention programs should encourage individuals to change behaviors due to self-determined motives [52]. Possible strategies for enhancing autonomous motivation are motivational interviewing [53] or the development of plans for changing behavior, such as implementation intentions [52]. Based on this research, the implementation intentions should specify motivational cues to decrease an unhealthy behavior instead of situational cues. For example, an implementation intention that focuses on motivational cues could be “If I feel social pressure (motivational cue) to use the elevator, then I will…” instead of “If I am with friends (situational cue) and feel I want use the elevator, then I will…” In line with research by Adriaanse *et al.* [52], the concentration on motivational cues (why; perceived reason) instead of situational cues (where/when) seems to be a promising approach for future research.

If interventions that target increased stair use involve decision prompts as an intervention strategy (e.g., [34]), based on our results, flyers or posters should be designed to address autonomous motivation of the targeted individuals. Thus, instead of focusing on external rewards such as “You can save energy by taking the stairs” or descriptive norms like “Cool people take the stairs,” slogans that focus on pleasure and personally relevant cues such as “You are a healthy person—take the stairs” are more worthwhile. The reason is that such slogans may enhance autonomous motivation. This should help initiate the healthy behavior of taking the stairs.

Furthermore, increased autonomous motivation might have a positive impact on CHBs as suggested by this study. It can cautiously be assumed that the boost of CHBs might have positive implications for an intended behavior change. However, the CHBs in our study comprised the intended behavior as compensatory behavior. Nevertheless, interventions could focus on CHBs because the belief that compensation for an unhealthy behavior is possible might facilitate a behavior change (*cf.* [43]). The perceived difficulty of engaging in the intended behavior might be decreased by the belief that failures to act against one’s intention can be compensated. Thus, promoting possible compensations of an unhealthy behavior, which in our study was physical inactivity, should foster health behaviors. Plans for balancing one’s own health behavior could be created (e.g., like the Weight Watchers program). However, further research is needed to examine whether CHBs promote a behavior change in the long run or undermine it, since the need for compensation may diminish over time. In addition, whether individuals tend to over-substitute unhealthy behavior with compensatory behavior should be investigated, since this could also imply negative health effects (e.g., excessive exercising, restrained eating).

As one of the first studies that has investigated the interplay of autonomous motivation and CHBs for readiness to change a behavior and the self-reported behavior itself, this study provides the first indications that to change health behaviors both psychological constructs should be used.
